# The capacity of *Aspergillus niger* to sense and respond to cell wall stress requires at least three transcription factors: RlmA, MsnA and CrzA

**DOI:** 10.1186/s40694-014-0005-8

**Published:** 2014-12-01

**Authors:** Markus RM Fiedler, Annett Lorenz, Benjamin M Nitsche, Cees AMJJ van den Hondel, Arthur FJ Ram, Vera Meyer

**Affiliations:** 1 Institute of Biotechnology, Department Applied and Molecular Microbiology Berlin University of Technology, Gustav-Meyer-Allee 25, Berlin, D-13355 Germany; 2 Institute of Biology Leiden, Leiden University, Molecular Microbiology and Biotechnology, Sylviusweg 72, Leiden, 2333 BE The Netherlands; 3 HiTeXacoat, Waterlelie 124, Gouda, 2804 PZ The Netherlands; 4 Kluyver Centre for Genomics of Industrial Fermentation, Delft, 2600 GA The Netherlands

**Keywords:** Cell signaling, Cell wall, Chitin, Drug action, Fungi, Transcriptomics

## Abstract

**Background:**

Cell wall integrity, vesicle transport and protein secretion are key factors contributing to the vitality and productivity of filamentous fungal cell factories such as *Aspergillus niger*. In order to pioneer rational strain improvement programs, fundamental knowledge on the genetic basis of these processes is required. The aim of the present study was thus to unravel survival strategies of *A. niger* when challenged with compounds interfering directly or indirectly with its cell wall integrity: calcofluor white, caspofungin, aureobasidin A, FK506 and fenpropimorph.

**Results:**

Transcriptomics signatures of *A. niger* and phenotypic analyses of selected null mutant strains were used to predict regulator proteins mediating the survival responses against these stressors. This integrated approach allowed us to reconstruct a model for the cell wall salvage gene network of *A. niger* that ensures survival of the fungus upon cell surface stress. The model predicts that (i) caspofungin and aureobasidin A induce the cell wall integrity pathway as a main compensatory response via induction of RhoB and RhoD, respectively, eventually activating the mitogen-activated protein kinase kinase MkkA and the transcription factor RlmA. (ii) RlmA is the main transcription factor required for the protection against calcofluor white but it cooperates with MsnA and CrzA to ensure survival of *A. niger* when challenged with caspofungin and aureobasidin A. (iii) Membrane stress provoked by aureobasidin A via disturbance of sphingolipid synthesis induces cell wall stress, whereas fenpropimorph-induced disturbance of ergosterol synthesis does not.

**Conclusion:**

The present work uncovered a sophisticated defence system of *A. niger* which employs at least three transcription factors - RlmA, MsnA and CrzA – to protect itself against cell wall stress. The transcriptomic data furthermore predicts a fourth transfactor, SrbA, which seems to be specifically important to survive fenpropimorph-induced cell membrane stress. Future studies will disclose how these regulators are interlocked in different signaling pathways to secure survival of *A. niger* under different cell wall stress conditions.

**Electronic supplementary material:**

The online version of this article (doi:10.1186/s40694-014-0005-8) contains supplementary material, which is available to authorized users.

## Background

Fungi are among the most serious biological threats to humans and plants. They kill as many people as tuberculosis and malaria and spoil about 10% of the world’s annual harvest [1],[2]. Although several antifungal drugs are available, their success is limited due to toxicity, a narrow spectrum of activity, detrimental drug interactions and the development of drug resistance [2]. To mitigate this public threat, safe and effective antifungal drugs are therefore needed. Key to this is a better understanding on how fungi sense and respond to antifungal drugs.

A preferred target for new antifungal drugs is the fungal cell wall as its composition is fundamentally different from bacterial and plant cell walls. In addition, the cell wall from yeast and filamentous fungi display significant architectural differences which can potentially be exploited. Whereas β-1,3-glucans, β-1,4 glucans, β-1,6-glucans, chitin, chitosan and glycoproteins are major constituents found in both, α-1,3-glucans and galactomannans are specific for filamentous fungi. Furthermore, chitin is much more abundant in cell walls of filamentous fungi [3]-[6]. At least three signaling pathways have been shown to be involved in the cell wall compensatory response in the yeast model *Saccharomyces cerevisiae* when confronted with cell wall disturbing compounds: the Pkc1p-Slt2p signaling pathway (also named cell wall integrity (CWI) pathway) mediated by the transcription factors Rlm1p and Swi4p/Swi6p, the general stress response pathway mediated by Msn2p/Msn4p, and the calcium/calcineurin pathway mediated by Crz1p [7],[8]. Whereas cell wall stress responses are well studied and understood in *S. cerevisiae*, much less is known from *Aspergilli*, a genus comprising many human and plant pathogenic filamentous fungi. Components of the CWI signaling cascade, the general stress response pathway and the calcium/calcineurin pathway are, however, conserved among *Aspergilli* [9]-[13].

Using *A. niger* as a model system, we recently studied its defense strategies against cell-surface acting compounds such as caspofungin (CA, inhibitor of β-1,3-glucan synthesis), fenpropimorph (FP, inhibitor of ergosterol synthesis), the antifungal protein AFP (inhibitor of chitin synthesis) and calcofluor white (CFW, inhibitor of chitin microfibril assembly) [9],[12],[14]. Common to these compounds is that they induce the CWI pathway in *A. niger* as compensatory response. By activation of this signaling pathway, cell wall reinforcing genes such as the *agsA* gene (encoding α-1,3-glucan synthase) become transcriptionally activated through the RlmA transcription factor, the ortholog of the *S. cerevisiae* Rlm1p protein [12],[14],[15]. Most surprisingly, this cell wall salvage mechanism is sufficient to ensure survival of *A. niger* when subjected to CFW [12] but not when stressed with the antifungal protein AFP. Although both compounds target cell wall chitin and induce expression of RlmA and its effector genes via the CWI pathway, this defense strategy is not the most appropriate one to protect *A. niger* against AFP. Instead, triggering the calcium/calcineurin signaling pathway which in turn induces expression of the chitin synthase gene *chsD* confers a higher protection to *A. niger* against AFP [9]. These observations suggest that the CWI pathway of *A. niger* is, as in *S. cerevisiae*, not the only compensatory mechanism important for the repair of compromised cell walls.

To obtain a more comprehensive view on the cellular responses that allow *A. niger* to adapt to and survive to cell wall stress conditions, we characterized in this study its transcriptional adaptation program when stressed with the calcium/calcineurin signaling inhibitor FK506 and with the inhibitor of sphingolipid synthesis aureobasidin A (AbaA). Block of sphingolipid synthesis by AbaA has been shown to trigger protein kinase C signaling resulting in activation of Slt2p, the terminal MAP kinase of the CWI pathway in *S. cerevisiae* [16]. The experimental approach of the present study was similar to that of our previous work, where we determined the transcriptomic fingerprint of *A. niger* when stressed with CA and FP, respectively [14]. The identical approach allowed us to directly compare the data from both studies and enabled the identification of general survival and antifungal-specific stress responses. In brief, young germlings of *A. niger* were cultivated in bioreactors to ensure controlled, reproducible and equal growth conditions. Antifungals were added at sublethal concentrations that permitted *A. niger* to adapt to the growth-inhibitory effects and to respond with the formation of new (sub)apical branches. We also determined the transcriptomic and phenotypic consequences of inactivating the *rlmA* gene in *A. niger*. Overall, our transcriptomic data led to the conclusion that in addition to RlmA, MsnA, a predicted Msn2p orthologue, as well as CrzA are important for *A. niger* to withstand cell wall stress conditions. We therefore characterized the function of MsnA and CrzA for cell survival of *A. niger* by analyzing respective null strains.

## Results

*Survival responses of* A. niger *against AbaA and FK506.* Spores of the *A. niger* wild-type strain N402 were allowed to germinate after which they were treated with different concentrations of the sphingolipid biosynthesis inhibitor AbaA and the inhibitor of calcium/calcineurin signaling FK506. As previously reported, sublethality provokes a morphological response of *A. niger* germlings, including the formation of (sub)apical branches thereby reflecting an adaptation program of *A. niger* to the cell wall stress condition [14]. We therefore determined sublethal concentrations of AbaA and FK506 and studied the morphological responses of *A. niger* germlings in a small scale format (5 ml, data not shown). Based on this preliminary experiment, we determined that sublethal concentrations for the bioreactor scale are 2 μg/ml for AbaA and 1.28 μg/ml for FK506, respectively (working volume 500 ml). Here, 1 × 10^6^ spores/ml were cultivated in Fermentation medium (FM), whereby the dissolved oxygen tension was used as an indicator for equal growth conditions in the different bioreactor runs (data not shown). *A. niger* spores were allowed to germinate for 5 h, after which 2 μg/ml AbaA or 1.28 μg/ml FK506 were added. After an additional hour of cultivation, samples were taken to microscopically determine the BI-value, (giving the percentage of germlings displaying (sub)apical branches (n > 100)) and to isolate RNA for transcriptomic analyses. As displayed in Table 1, the addition of AbaA and FK506 increased the BI value about 4-fold and 2-fold, respectively, whereby both compounds also significantly inhibited germ tube elongation.

**Table 1:** **The effect of AbaA (2 μg/ml) and FK506 (1,28 μg/ml) on** ***A. niger*** **germ tube formation and elongation**

Sample	BI (%)	Mean BI (%)	Polarity axes (%)			Mean length (μM)
			N = 1	N = 2	N = 3	
NC^AbaA^	2	3 ± 1	89	10	1	20.9 ± 0.9
	2					
	3					
AbaA	14	13 ± 1	77	20	3	14.4 ± 0.1
	13					
NC^FK506^	5	5 ± 1	93	7	0	18.8 ± 1.8
	4					
FK506	12	11 ± 3	73	24	4	11.7 ± 0
	9					

At least *A. niger* 100 germlings were counted per single fermentation run to analyze the number of germ tubes present. From each two or three independent fermentation runs, a mean BI was calculated. AbaA, cells treated with aureobasidin A; FK506, cells treated with FK506; NC^AbaA^, cells neither treated with AbaA or FK506, negative control for AbaA (solvent ethanol); NC^FK506^, cells neither treated with AbaA or FK506, negative control for FK506 (solvent DMSO). N = number of germ tubes per germling.

RNA samples extracted from duplicate cultures of AbaA-, FK506-, and nontreated samples were used for transcriptomic comparison. Raw chip data was normalized together with our previously published microarray data for CA- and FP-treated samples [14] to allow direct comparison (see Methods). Differential gene expression was evaluated by moderated t-statistics using a false discovery rate (FDR) < 0.05. Note that a minimal fold-change criterion was not applied for the identification of differentially expressed genes, as fold-changes not necessarily relate to biological relevance [17],[18]. The complete list of differentially expressed genes of AbaA-, FK506-, CA-, FP- treated samples including fold change and statistical significance is given in the Additional file 1 and Additional file 2. A total of 236, 96, 141, and 24 genes out of 13,989 *A. niger* genes were identified as differentially expressed upon exposure to AbaA, FK506, CA, and FP. Most of the responsive genes were up-regulated (179, 49, 139 and 22). The differentially expressed genes were grouped according to FunCat [19] (Table 2) and the predicted protein functions [20] revisited by BlastP analysis (Additional files 3, 4, 5 and 6).

**Table 2:** **FunCat classification of** ***A. niger*** **genes responsive to the treatment with caspofungin (CA), aureobasidin A (AbaA), FK506 and fenpropimorph (FP)**

Functional category	AbaA	FK506	CA ^a^	FP ^a^
Sum of differentially expressed genes	236	96	141	24
Number of up-regulated genes	179	49	139	22
Number of down-regulated genes	57	47	2	2
Metabolism	42	11	26	16
Energy	2	2	2	-
Cell cycle and DNA processing	6	-	-	-
Transcription	14	3	3	-
Protein synthesis	4	-	1	-
Protein fate	10	7	23	1
Protein with binding function or cofactor requirement	3	1	-	-
Cellular transport, transport facilitation and transport routes	16	8	13	1
Cellular communication/signal transduction mechanism	5	1	4	1
Cell rescue, defense and virulence	3	3	4	-
Interaction with the environment	1	6	-	-
Cell fate	3	-	-	-
Biogenesis of cellular components	4	-	2	-
Unclassified proteins	123	54	63	5

An annotated list of all genes, including fold change, *p*- and *q*-value and classification can be found in Additional file 2. ^a:^ microarray data from [14].

**Figure 1:**
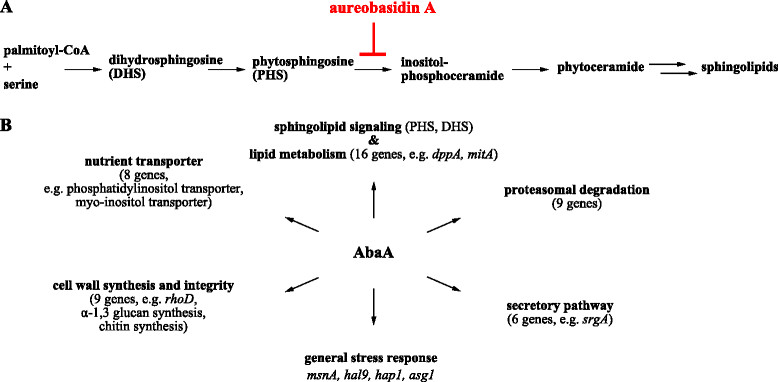
**The transcriptomic response of** ***A. niger*** **towards aureobasidin A. (A)** Sphingolipid synthesis pathway and the site of action of aureobasidin A (AbaA). **(B)** Selected aureobasidin A responsive genes clustered into different functional categories (based on Additional file 3).

*Transcriptome response of A. niger to aureobasidin A.* The cyclic depsipeptide aureobasidin A interferes with fungal sphingolipid metabolism by blocking the Golgi-localized enzyme inositolphosphorylceramide (IPC) synthase [21], which synthetizes inositol phosphoceramide, a main precursor for sphingolipids. AbaA-induced block of sphingolipid synthesis in *S. cerevisiae* has multiple consequences: the transport of proteins from the ER to the plasma membrane is inhibited, the functionality of nutrient transporters at the plasma membrane as well as plasma membrane integrity is compromised, and actin assembly as well as chitin deposition are disturbed [22],[23]. AbaA therefore activates the MAP kinase Slt2p of the CWI pathway and the TORC2-Slm1/2p-Ypk1p-Orm1/2p cascade as compensatory responses in *S. cerevisiae* [24]-[26]. The transcriptome data suggest that similar processes were affected in *A. niger* germlings when stressed with AbaA: proteins predicted to function in (i) lipid biosynthesis, (ii) vesicle transport from the ER to the Golgi, (iii) chitin, α-1,3-glucan and β-1,3-glucan synthesis and remodeling, (iv) actin polarization and (v) nutrient transport were significantly up-regulated in AbaA-treated *A. niger* cells (Figure 1A and B and Additional file 3). Worth highlighting are the following ORFs as they are predicted to function as regulatory proteins: (i) An02g01180, a predicted diacylglycerol pyrophosphate phosphatase and ortholog of Dpp1p, which synthesizes the secondary messenger diacylglycerol (DAG), the activator of mammalian and fungal protein kinase C of the CWI pathway [27], (ii) An02g08000, a predicted G-protein alpha subunit (Gpa2p ortholog) important for cAMP-protein kinase A signaling in *S. cerevisiae* and activator of the transcription factors Msn2/4p [7], (iii) An07g05960, encoding the Msn4p ortholog, (iv) RhoD (Rho4p ortholog), encoding a Rho GTPase important for cell wall integrity, septum formation and CFW resistance in *A. niger* [13], (v) SrgA (Sec4p ortholog), a Rab GTPase important for secretion, polarity maintenance and CA resistance of *A. niger* [14],[28], (vi) An13g01040, a CA-responsive gene of *A. niger* [14] encoding a putative Rab-geranylgeranyltransferase (Bet2p ortholog), which provides a membrane attachment moiety for Sec4p thus ensuring vesicle transport between ER and Golgi in *S. cerevisiae* and (vii) An05g01070, the ortholog of Rsb1p, a flippase that transports the sphingolipid signaling molecules phytosphingosine (PHS) and dihydroxysphingosine (DHS) across membranes of *S. cerevisiae* [29]. Both PHS and DHS are located upstream of IPC in the sphingolipid biosynthesis pathway and activate ubiquitin-dependent proteasomal protein degradation during heat stress-induced transient cell cycle arrest in *S. cerevisiae* [30],[31]. As expression of eight *A. niger* genes predicted to function in proteasomal protein degradation were also up-regulated upon AbaA treatment (Figure 1B and Additional file 3) suggests that the mode of action of AbaA as well as the cellular function of sphingolipids might be similar in *S. cerevisiae* and *A. niger*. Finally, the transcriptomic signature of *A. niger* also pointed towards a general stress response of *A. niger* upon AbaA treatment, because orthologs to stress-related transcription factors of *S. cerevisiae* such as Asg1p (An07g07050), Hap1p (An15g02080) and Hal9p (An12g09020) displayed significant up-regulation.

**Figure 2:**
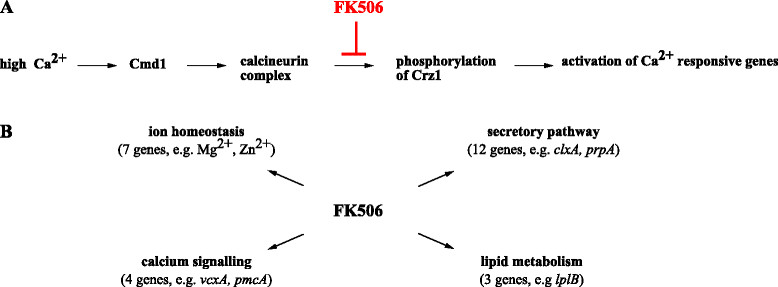
**The transcriptomic response of** ***A. niger*** **towards FK506. (A)** Schematic overview of the calcineurin singalling pathway and the site of action of FK506. **(B)** Selected FK506 responsive genes clustered into different functional categories (based on Additional file 4).

*Transcriptome response of A. niger to FK506.* FK506 is a macrolide lactone that blocks calcium/calcineurin signaling in *S. cerevisiae* in response to high cytosolic calcium levels by inhibiting the protein phosphatase calcineurin. Calcineurin is an activator of the transcription factor Crz1p, which drives expression of more than hundred genes including genes that encode the Ca^2+^ pumps Pmc1p and Pmr1/2, the β-1,3-glucan synthase Fks2p or other transcription factors such as Hal1p [32]. Additionally, calcineurin can repress the activity of proteins, including the vacuolar calcium exchanger Vcx1p, in a Crz1p-independent manner [33]. In contrast, CrzA, the Crz1p ortholog of *A. nidulans* is a positive regulator of the *vcxA* gene (*VCX1* ortholog) and *chsB* encoding a chitin synthase [34]. Whereas the function of CrzA has not been studied yet in *A. niger*, it was demonstrated that elevated calcium levels induce expression of the chitin synthase encoding gene *chsD* [9]. Consistent with a role of CrzA for calcium homeostasis in *A. niger* is the observation that treatment of *A. niger* germlings with FK506 provoked up-regulation of ORFs encoding VcxA homologs (An19g00340, An19g00330, An19g00320) and a predicted calcium pump (PmcA, An19g00350). In addition, expression of other ion transporters were affected as well including predicted Mg^2+^ and Zn^2+^ transporters (An01g01950, An05g00640, An12g10320) (Figure 2A and B and Additional file 3).

Calcium has a regulatory function for several transporting steps in the constitutive secretory pathway in eukaryotes [35]. Congruently, twelve genes predicted to function in protein folding, protein maturation and vesicle trafficking were differentially expressed upon FK506 treatment of *A. niger* germlings, eleven of which displayed increased expression and nine of which are also responsive genes to the treatment of *A. niger* with the ER-stressing agents DTT and tunicamycin [36], e.g. genes involved in signal peptide cleavage (An09g05420), protein translocation from and to the ER (An03g04940, An02g01510, An08g00740), glycosylation and quality control (An15g03330, An03g04410, An02g14930) or functioning as chaperones (An01g08420/ClxA and An01g04600/PrpA). Surprisingly, none of the FK506-responsive genes could be directly attributed to cell wall remodeling although two predicted G-protein coupled receptors were differentially expressed (An04g02930 and An02g01560/GprD). GprD shows similarity to the human LPA_2_ (EDG4) protein which acts as specific receptor for lysophosphatidic acid to activate calcium signaling and downstream protein kinase C [37]. Supportively, GprD has been predicted to integrate stress signals via the calcineurin pathway in *A. fumigatus* [38] which, however has not been observed in *A. nidulans* [39].

**Figure 3:**
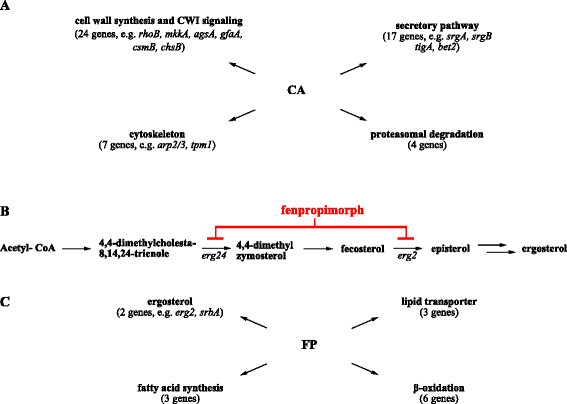
**The transcriptomic response of** ***A. niger*** **towards caspofungin and fenpropimorph. (A)** Selected caspofungin (CA) responsive genes clustered into different functional categories (based on Additional file 5). **(B)** Schematic overview of the ergosterol synthesis pathway and the site of action of fenpropimorph (FP). **(C)** Selected fenpropimorph responsive genes clustered into different functional categories (based on Additional file 6).

*Transcriptome response of A. niger to caspofungin.* Our transcriptomic fingerprint of CA-treated *A. niger* germlings has been published previously [14]. However, we decided to revisit the dataset using a false discovery rate FDR of 0.05 as it is a more stringent cut-off criterion compared with the fold change criterion of 1.5. The rationale behind was to perform a statistically more significant analysis and to directly compare the transcriptomic signature of CA-treated germlings with the datasets of AbaA- and FK506-treated cells for the identification of commonly regulated genes. Basically, the CA-responsive dataset changed from 172 genes [14] to 141 genes (this study). Figure 3A and Additional file 5 highlight key processes and genes which ensured survival of *A. niger* upon CA treatment. In brief, CA likely provoked a remodeling of *A. niger* cell walls by stimulating transcription of members of the CWI signaling pathway: RhoB (regulator of α-1,3 glucan synthase), An10g00490 (Rho-GAP), An18g04590 (Rho-GDP dissociation inhibitor) as well as the protein kinase kinase MkkA. Consistently, 20 genes functioning in cell wall synthesis and remodeling were up-regulated including genes involved in chitin, α-1,3-glucan and β-1,6-glucan synthesis as also identified previously [14]. The secretory pathway and cytoskeleton are fundamental in transport of cell wall synthetizing enzymes to the hyphal tip. 17 genes belonging to the secretory pathway were congruently up-regulated (Additional file 5), among which were the GTPases SrgA and SrgB as well as An13g01040, a predicted geranlygeranlytransferase and ortholog of Bet2p, which geranylates several secretory pathway GTPases in *S. cerevisiae* [40]-[42]. Likewise, seven genes encoding proteins involved in cytoskeleton organization and polarization displayed increased expression values, e.g. the Arp2/3 complex (An01g05510, An18g06590, An08g06410) and tropomyosin (An13g00760) to name but a few.

Previously not identified was a group of four genes coding for proteins having a function in proteasomal degradation, i.e. An18g06700 (Pre7p ortholog), An18g06680 (Pre4p ortholog) and An04g01870 (Pre1p ortholog) and An14g00180 (Rpt6p ortholog; Additional file 5).

*Transcriptome response of A. niger to fenpropimorph*. FP is an inhibitor of ergosterol biosynthesis in *S. cerevisiae* by inhibiting sterol C-14 reductase (*ERG24* gene) and sterol C-8 isomerase (*ERG2* gene) [43]. Expression of only 24 genes was modulated in *A. niger* upon FP treatment, among which was the predicted Erg2p ortholog, which suggests that FP exerts a similar mode of action in *A. niger*. (Figure 3B and C and Additional file 6). 14 of the responsive genes are indeed predicted to function in lipid metabolism and were all up-regulated upon FP stress: (i) SrbA, a transcription factor shown to control iron and ergosterol homeostasis in *A. fumigatus* [44], (ii) genes involved in β-oxidation (An08g05400, An17g01150, An15g01280, An08g07520, An16g04520, An14g00990), (iii) genes involved in fatty acid biosynthesis (An16g05340, An07g03290, An15g02830) and (iv) genes predicted to function in lipid transport across the peroxisomal, mitochondrial and plasma membrane (An18g01590, An04g00740, An01g12380). Hence, remodeling plasma membranes via lipid degradation and *de novo* synthesis might be the most appropriate compensatory response of *A. niger* to withstand FP-mediated inhibition of ergosterol homeostasis. In agreement, pyruvate carboxylase (An15g02820), a protein fueling the Krebs cycle was up-regulated, possibly reflecting the higher need of acetyl-CoA for fatty acid biosynthesis.

**Figure 4:**
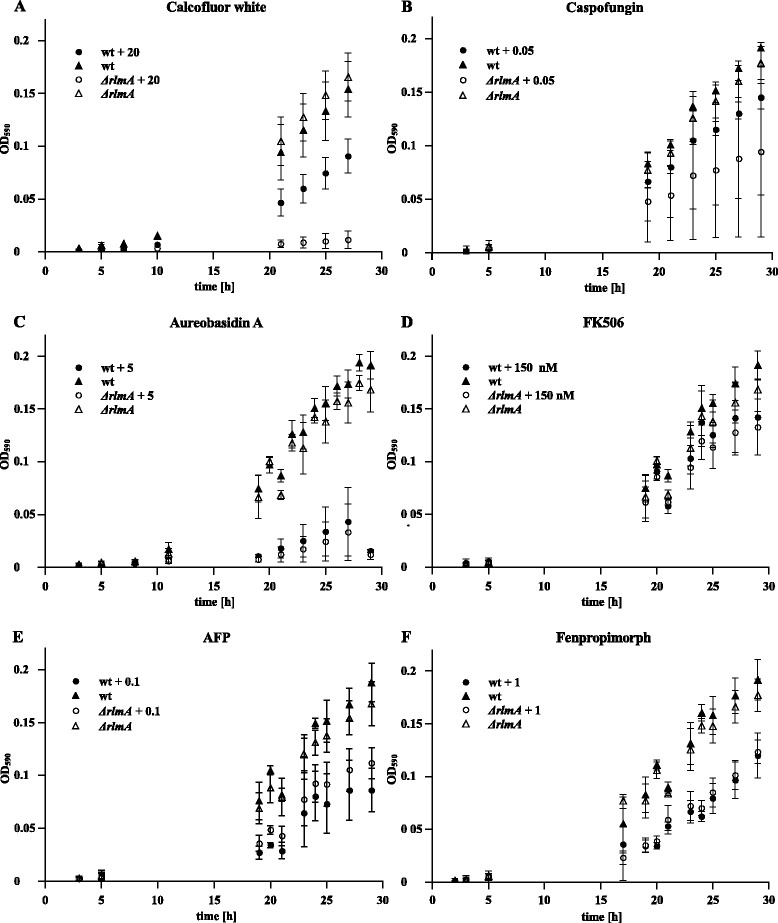
**The role of RlmA in counteracting antifungals.** 10^4^ spores of strains N402 (filled symbols) and MA47.1 (empty symbols) were cultivated in 200 μl MM medium supplemented with 0.003% yeast extract for 30 h at 30°C in microtiter plates in the presence (●) or absence (▲) of different antifungals. The data for calcofluor white, caspofungin, aureobasidin A, FK506, AFP and fenpropimorph are shown in panel **A, B, C, D, E** and **F,** respectively. For each compound, the same volume of its solvent was used for the negative control. The final concentration of the compounds is indicated in the legend and given in μg/ml.

*The role of RlmA in the defense response against cell surface stress*. We have previously shown that the survival response of *A. niger* against the cell wall disturbing compounds CFW, CA, FP and AFP involves up-regulation of the α-1,3-glucan synthase AgsA, which is mediated by the activity of the transcription factor RlmA [14],[15]. In order to test whether RlmA is of general importance for the defense against these four antifungals as well as AbaA and FK506, liquid growth inhibition assays were performed using the *rlmA* deletion strain MA47.1 [12] and the wild type strain N402 as test strains (see Methods). To our surprise, the *ΔrlmA* strain was only susceptible towards the chitin inhibitor CFW but not towards the other five compounds (Figure 4), suggesting that RlmA might not be the only transcription factor necessary to counteract cell wall and cell membrane stress.

**Figure 5:**
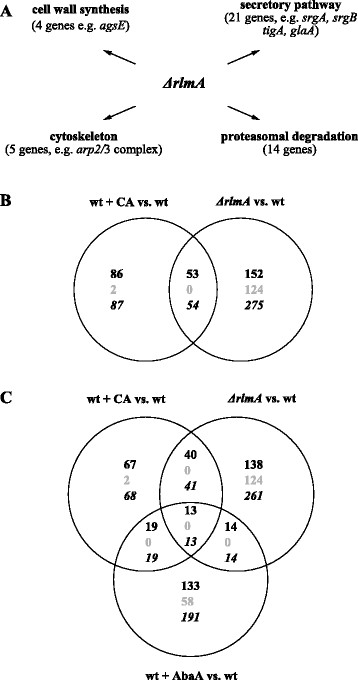
**Deletion of** ***rlmA*** **provokes a stress response which is similar to treatment of the wild-type with caspofungin. (A)** Responsive genes to *rlmA* deletion clustered into different functional categories (based on Additional file 7). **(B and C)** Venn diagrams of induced (black), repressed (grey) and up- or down-regulated (italic) genes for the wild type treated with caspofungin versus the wild type (wt + CA vs. wt), the *∆rlmA* strain versus the wild-type (*∆rlmA* vs. wt) and the wild-type treated and non-treated with aureobasidin A (wt + AbaA vs. wt).

To investigate the RlmA loss-of-function phenotype in more detail, the transcriptomic signatures of the *ΔrlmA* strain was compared with that of the wild-type. Both strains were allowed to germinate in bioreactor cultivations as described above but were not stressed with any antifungal compound. After a total cultivation of 6 h, samples were taken for RNA extraction and microarray hybridization. In total, 329 genes were found to be differentially expressed, whereby 205 displayed increased and 124 genes decreased expression in the *ΔrlmA* strain when compared with the wild-type strain (Additional file 2). This transcriptomic fingerprint included genes involved in cell wall remodeling (4 genes), protein secretion (21 genes), cytoskeleton remodelling (5 genes), proteasome function (14 genes) and vacuolar integrity (4 genes, Figure 5A and Additional file 7). Most interestingly, 54 of the 329 responsive genes in the *ΔrlmA* strain were overlapping with the responsive gene set of the wild-type strain when stressed with CA (Figure 5B). The complete set of overlapping genes is provided in Additional file 8. Some of the up-regulated genes are briefly summarized as follows: the secretion-related GTPases SrgA and SrgB, the protein disulfide isomerase TigA, a subunit of the SEC61 complex (An01g11630), the ER dolichyl-phosphate manosyltransferase DpmA, subunits of the actin nucleation complex Arp2/3 (An01g05510, An18g06590), tropomyosin TpmA, the tubulin-specific chaperone TbcA and proteasomal degradation proteins (An18g06700, An14g00180, An18g06680). Overlapping and up-regulated in the CA-treated wild-type but down-regulated in the *ΔrlmA* strain was the α-1,3-glucan synthase AgsE, thus strongly indicating that this gene is a direct target of RlmA and its regulation is merely RlmA-dependent. Interestingly, deletion of the *rlmA* gene also caused down-regulation of An18g05830, the predicted nuclear pore protein karyopherin Kap121 ortholog, known to import stress-related transcription factors in *S. cerevisiae* such as Pdr1p (pleiotropic drug resistance), Aft1p (iron depletion, DNA replication stress) and Yap1p (oxidative stress) [45]-[47].

This dataset and its comparison with that of the wild-type led us to two hypotheses: First, RlmA is not the only cell wall stress related transcription factor as deletion of RlmA causes only down-regulation of one cell wall gene (*agsA*), whereas CA-induced stress in the wild-type induced expression of 21 cell wall genes. Second, inactivation of RlmA could have provoked a compensatory response in *A. niger* which confers a strong cross protection against cell wall stressors. In favor of the latter hypothesis are three observations: (i) Genes involved in cell wall remodeling, protein secretion and actin polarization were up-regulated in the deletion strain (Figure 5A and Additional file 7). (ii) The *ΔrlmA* strain did not show any hypersensitivity against CA, FP, AFP, FK506 and AbaA (Figure 4). (iii) Cultivation of the deletion strain in bioreactor settings and treatment with 50 ng/ ml CA did not provoke any significant transcriptomic response (data not shown), whereas it did for the wild-type strain (see above). It might be conceivable, however, that higher concentrations of CA would provoke a transcriptomic response in the *ΔrlmA* strain.

*Promoter analysis of differentially expressed genes.* In order to identify transcription factors, which in addition to RlmA mediate the survival response of *A. niger* to the cell surface stressors CA, FP, AbaA and FK506, we screened the 1,000-bp upstream regions of all differentially expressed genes for the presence of binding sites established for 25 transcription factors from different *Aspergillus* and *Trichoderma* species [48] and determined whether these motifs were significantly over- or underrepresented (500,000 bootstrap samples, FDR < 0.05). Binding sites for the transcription factors RlmA and MsnA were significantly enriched in the CA-responsive gene set of the wild-type strain, implying that MsnA could play in addition to RlmA an important role for the resistance of *A. niger* against cell wall stress while no binding sites were significantly enriched for either AbaA or FK506 treatment.

**Figure 6:**
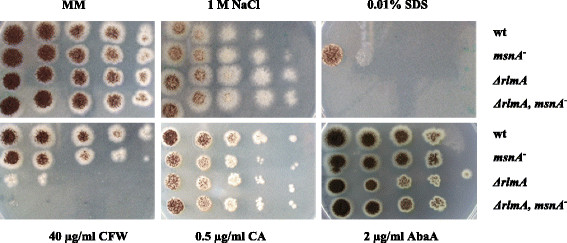
**Plate sensitivity assays of** ***rlmA*** **and** ***msnA*** **null mutants towards different stress conditions.** 5 μl of a series of a tenfold dilution started with 10^7^ conidia per ml were spotted on MM plates supplemented with the indicated amounts of NaCl, SDS, caspofungin (CA), calcofluor white (CFW) and aureobasidin A (AbaA), respectively and incubated at 30°C for 3 days.

*Phenotypic analyses of MsnA and RlmA deletion strains.* MsnA has been shown to be positively involved in oxidative stress response, osmotic stress-coupled maintenance of polar growth and secondary metabolism in different *Aspergilli* [49]-[51]. In *S. cerevisiae*, the orthologs Msn2/4p are involved in several stress responses, including heat shock, osmotic shock, oxidative stress and pH resistance [52]. However, Msn2/4p are dispensable for the stress response in the yeast *Candida albicans* [53]. To unravel the function of MsnA for cell wall stress survival of *A. niger*, a *msnA* disruption strain and a *ΔrlmA, msnA*^*−*^ double mutant strain were generated and their phenotypes on solid media compared with the wild-type strain and the *ΔrlmA* deletion strain. Interestingly, all three mutant strains were not hypersensitive towards high osmolarity (2% glycerol), high temperature (42°C), high pH (pH 9.0), oxidative stress (4 mM H_2_O_2_), the presence of AFP (0.2 μg/ml), FP (1 μg/ml) or FK506 (10 μM; data not shown). However, different sensitivities were observed when the strains were treated with CFW, CA, AbaA, SDS or subjected to NaCl-mediated salt stress (Figure 6). A mildly increased tolerance of the single and double *rlmA* deletion strains were observed towards 1 M NaCl, suggesting that increased expression and secretion of cell wall genes and actin cytoskeleton might confer a tolerance towards high salt stress. In *S. cerevisiae*, it was shown that cell wall remodeling is indeed part of its physiological response to withstand salt stress [54],[55]. In return, the *msnA* knockout strain displayed a substantial higher tolerance towards the surface-active anionic detergent SDS when compared to the wild-type. This tolerance was interestingly not observed in the *ΔrlmA* strain and was abolished in the double mutant strain (Figure 6), suggesting that the RlmA-mediated response towards SDS-induced membrane stress is epistatic over the MsnA-dependent response or that remodeling of the cell wall in the *ΔrlmA* strain may prevents any MsnA-mediated response. It is not completely surprising that loss of MsnA can confer higher tolerance to some compounds. For example, deletion of Msn2p also caused reduced sensitivity towards some cell wall stressing agents [56]. As SDS induces the pleiotropic drug-resistance network in *S. cerevisiae*, thought to pump SDS and other small molecules out of yeast cells [57], it might be conceivable that loss of MsnA function in *A. niger* somehow also contributed to better extrusion of SDS out of the cells.

All three mutant strains became more sensitive towards CA, AbaA and CFW when cultivated on solid media (Figure 6), demonstrating that MsnA has - beside RlmA - a function in cell wall protection for *A. niger*. Whereas the contribution of both transcription factors was only very subtle with respect to 0.5 μg/ml CA and 2 μg/ml AbaA (note the *ΔrlmA* strain is insensitive to CA and AbaA when cultivated in liquid medium), both protect *A. niger* substantially against the chitin inhibitor CFW (40 μg/ml). Clearly, RlmA is the main contributor in the latter case because its inactivation resulted in a stronger growth-inhibited phenotype than inactivation of MsnA did. However, both seem to function in an additive or even synergistic manner, as the double mutant strains displayed a lethal phenotype on CFW plates. Notably, such an additive or synergistic phenotype was not observed on CA or AbaA plates, suggesting that both RlmA and MsnA function in the same signaling pathway when β-1,3-glucan polymerization or sphingolipid biosynthesis became inhibited.

**Figure 7:**
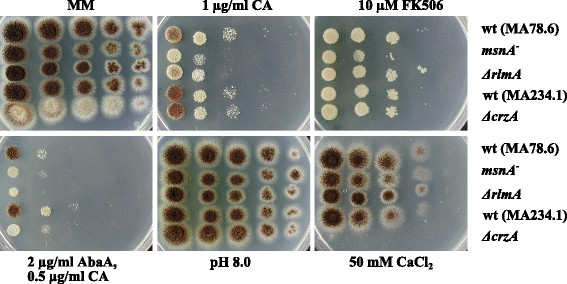
**Plate sensitivity assays of** ***crzA*** **,** ***rlmA*** **and** ***msnA*** **null mutants towards different stress conditions.** 5 μl of a series of a tenfold dilution started with 10^7^ conidia per ml were spotted on MM plates supplemented with the indicated amounts of CaCl_2_, caspofungin (CA), aureobasidin A (AbaA), FK506 and 100 mM MES to adjust pH 8, respectively and incubated at 30°C for 3 days.

*Phenotypic analyses of a* crzA *deletion strain.* The data above clearly showed that RlmA and MsnA are not the only transcription factors which protect *A. niger* against CA-induced cell wall stress. We thus rescreened the upstream regions of the CA-responsive gene set for the presence of enriched potential transcription factor binding sites. Indeed, when we lowered the length of the analyzed regions down to 500 bp instead of 1000 bp, CrzA binding sites appeared to be significantly enriched (63 genes out of 199 genes contain at least one CrzA binding site, FDR < 0.029; data not shown). To test whether CrzA might secure cell wall robustness as well, we analyzed the phenotype of a *crzA* null strain of *A. niger* (Ram et al., unpublished data) in the presence of CA, AbaA, FK506, CaCl_2_ and pH 8. Notably, the *crzA* deletion strain did not became hypersensitive towards FK506 and alkaline pH, which is in sharp contrast to the FK506- and pH 8 sensitive phenotype of the *A. nidulans* CrzA homologs [58]. In addition, the deletion strain was not hypersensitive towards CA and AbaA, respectively (Figure 7 and data not shown). However, CrzA is important for *A. niger* to withstand calcium-induced stress as well as the combined inhibitory effects of CA and AbaA (Figure 7). These observations corroborate the *in silico* data and demonstrate that CrzA has a minor but measurable function for *A. niger* to counteract cell wall stress.

**Figure 8:**
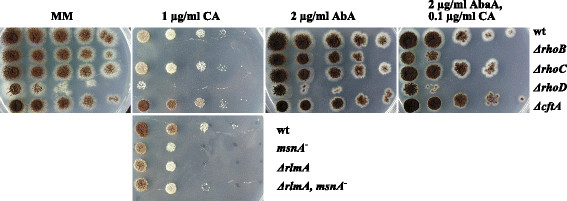
**RhoB and RhoD are important for the response of** ***A. niger*** **towards caspofungin and aureobasidin A. (A)** Sensitivity of the wild-type (wt) and *rhoB*, *rhoC*, *rho* or *cftA* deletion strain and **(B)** N402 (wt), JH1.1 (*msnA*^−^), MF3.2 (*ΔrlmA*) and MF4.10 (*ΔrlmA, msnA*^−^) towards caspofungin and aureobasidin A. 5 μl of a series of a tenfold dilution started with 10^7^ conidia per ml were spotted on MM plates supplemented with the indicated amounts of caspofungin (CA) and aureobasidin A (AbaA) and incubated at 37°C for 3 days.

*Identification of upstream regulators ensuring cell wall integrity of* A. niger*.* We have recently characterized the function of all six Rho-GTPases in *A. niger* and demonstrated that deletion of RhoB and RhoD rendered *A. niger* hypersensitive towards CFW [13]. This sensitivity is likely linked to their function - RhoB is mainly important for fast germination and sporulation, RhoD controls septum formation and fast hyphal growth rate. In the present study, the transcriptomic fingerprint of *A. niger* uncovered that the *rhoB* gene is among the CA-responsive genes and *rhoD* among the AbaA-responsive ones (Figures 1B and 3A). We thus performed additional growth-plate inhibition assays with the antifungal compounds used in this study (CA, AbaA, FP, FK506, SDS) to determine the tolerance of strains deleted for *rhoB* and *rhoD*, respectively. Strains deleted for the Rho-GTPase genes *rhoC* and *cftA*, which do not have a function in cell wall integrity [13], served as control strains in addition to the wild-type strain. No altered sensitivity of the mutant strains was observed when they became exposed to FP (1 μg/ml), FK506 (10 μM) and SDS (0.01%; data not shown); however, both *ΔrhoB* and *ΔrhoD* displayed increased sensitivity towards 0.5-1 μg/ml CA and 2 μg/ml AbaA (Figure 8 and data not shown). Notably, deletion of *rhoB* or *rhoD* rendered the strains even more sensitive towards CA than strains deleted for *msnA* or *rlmA*, implying that RhoB and RhoD target additional cell wall stress defense pathways in addition to the CWI pathway (eventually activating RlmA) and the pathway involving MsnA.

## Discussion

Reinforcement of the cell wall is an essential survival response to shield cells after exposure to distinct cell surface stressors. Fungi have therefore developed various signaling pathways which sense and transmit the stress signal to the cell interior and the nucleus which in turn modulates gene expression such that the cell responds most appropriately to the life threatening condition. The well-studied unicellular yeast *S. cerevisiae* has been used as the main model system to study the underlying mechanisms. It has evolved at least three signaling pathways - the CWI pathway with its central components Pkcp, Slt2p and Rlm1p, the general response pathway with its mediators cAMP, PKA and Msn2/4 and the calcium/calcineurin pathway with its main effectors calcineurin and Crz1p – to reinforce its cell wall by increasing chitin, glucan and cell wall protein levels. These pathways are interwoven to maintain cell wall integrity during growth-mediated cell wall expansion and to flexible react to osmotic and mechanic stress conditions [8].

The main modules of cell wall salvage pathways are genetically fixed in yeast and filamentous fungi such as *Aspergilli*. However, accumulating evidences suggest that the individual modules differ in their cellular assignment, although the architectural hierarchy and direction of signal transmission is similar. To name just a few examples: The MsnA transcription factor is crucial for the stress response in *S. cerevisiae* but *C. albicans* [53], sensors of the CWI pathway process differently stress signals in *A. fumigatus, A. nidulans, S. cerevisiae* and *Klyuveromyces lactis* [11],[59], the transcription factor CrzA does not act as activator of *VCX1* expression in *S. cerevisiae* but of *vcxA* expression in *A. nidulans* [34], the Rho GTPases RacA and CdcA/Cdc42 differ fundamentally in their function in *A. niger* and *A. nidulans* [13] and the exocyst-mediated vesicle transport of *S. cerevisiae* and *A. niger* is only partially conserved [26]. Hence, signal perception, transmission and translation can obviously differ among fungi which in fact could form a mechanistic explanation why fungi differ in their susceptibilities towards antifungal drugs. We have recently shown that the survival response of yeast and filamentous fungi towards the chitin synthase inhibitor AFP differs considerably. Whereas the presence of AFP provokes increased glucan synthesis via induction of the CWI pathway in AFP-sensitive fungi, AFP-resistant strains respond to AFP with enforced chitin synthesis by employing the calcium/calcineurin pathway [9]. Hence, the outcome of an antifungal attack strongly depends on the species-specific survival strategy chosen, which causatively might be linked to the different use of signaling pathways and their modules.

**Figure 9:**
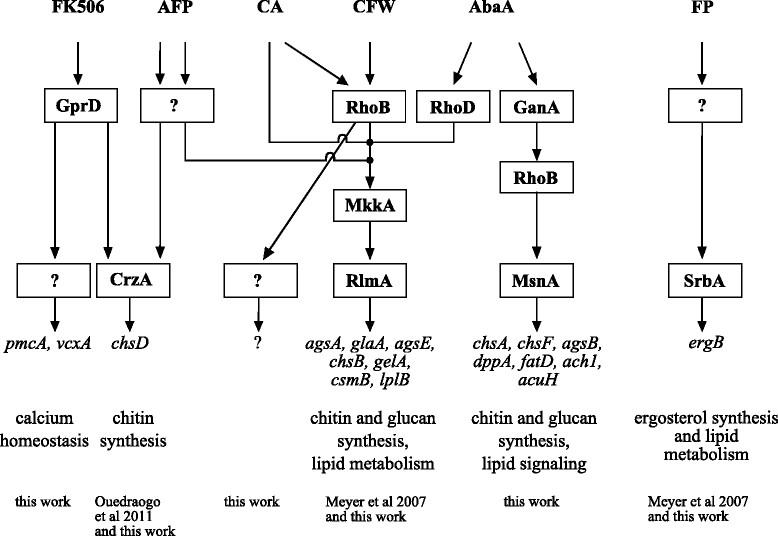
**Key players of the cell wall salvage gene network of** ***A. niger*** **deduced from transcriptomic and phenotypic analyses.** For details, see results and discussion.

**Figure 10:**
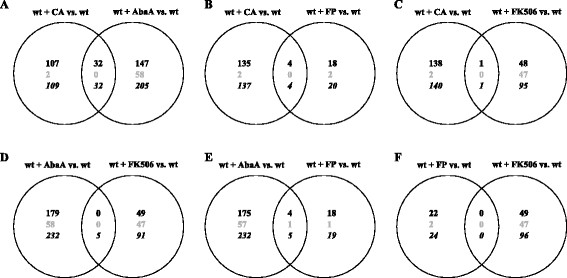
**Venn diagrams of genes induced by antifungals treatment.** Venn diagrams of absolute number of genes induced (black), repressed (grey) and up- or downregulated (italics) after treatment with aureobasidin A (AbaA), caspofungin (CA), fenpropimorph (FP) and FK506, respectively.

We are just at the beginning to understand cell wall stress survival strategies and the cross-talks between different signaling pathways specifically employed by *A. niger*, one of the main microbial production platforms in biotechnology. As cell wall integrity, vesicle transport, protein secretion and polarised growth are key factors contributing to the vitality and productivity of this cell factory, our efforts aim to decode the genetic basis of these processes and to understand how they are interlocked in order to pioneer rational strain improvement programs. The aim of the present study was thus to identify the transcriptomic signatures and physiological responses of *A. niger* when stressed with five different cell surface acting antifungals - CFW, CA, AbaA, FP and FK506. We used these signatures to predict regulator proteins mediating these responses and analysed the phenotypes of respective null mutant strains. This integrated approach allowed us to reconstruct a model for cell signaling pathways ensuring the survival of *A. niger* upon cell surface stress. The model is illustrated in Figure 9 and summarizes the following main conclusions: (i) Both inhibition of glucan synthesis via CA and inhibition of sphingolipid synthesis via AbaA induce the CWI pathway as a main compensatory response. The effector genes of this signaling route include chitin, glucan and (sphingo) lipid synthesizing genes. Key regulators of this response involve at least RhoB, RhoD, the protein kinase kinase MkkA and the transcription factor RlmA (Figures 1, 3A and 10 and Additional file 5). Such induced CWI pathway by CA and AbaA in *A. niger* is very well in agreement with observations made for *S. cerevisiae* [26], suggesting that cell wall integrity is not only strongly dependent on proper arrangement of cell wall polymers but also on sphingolipid homeostasis known to be important for cell membrane integrity and localization of cell wall proteins in *S. cerevisiae* [60]. Interestingly, the Venn diagram displaying overlapping CA and AbaA response genes (Figure 10A) predicts that even more genes are co-regulated by both drugs, suggesting that CA and AbaA stress signals are processed at least partially in similar signaling routes. (ii) Inhibition of ergosterol synthesis by FP does, however, not interfere with cell wall integrity (Figure 3B and Additional file 6) and only a few genes co-respond to both CA and FP treatment (Figure 10B). This suggests that ergosterol homeostasis is of somewhat minor importance for cell wall integrity and/or can be easier counteracted by post-transcriptional processes in *A. niger*. (iii) The activity of the transcription factor RlmA is crucially important for *A. niger* to survive treatments with the chitin inhibitor CFW. To a certain extent, RlmA also counteracts CA- and AbaA-induced stress (Figures 4, 8 and 9). The latter conclusion is corroborated by the fact that the *ΔrlmA* strain is unable to transcriptionally respond to treatments with CA, at least at concentrations which provoke cell wall stress in the wild-type situation (data not shown). Interestingly, lack of RlmA provokes a transcriptomic signature of *A. niger*, which is more than 50% identical to the wild-type’s signature when stressed with CA (Figure 5A, B and Additional file 7). This suggests that *A. niger* remodels its cell wall by the activity of additional transcription factor(s). (iv) Our *in silico* analyses of overrepresented transcription factor binding sites in the CA-responsive gene set disclosed MsnA and CrzA as potential candidate regulators. Indeed, single inactivation of the *msnA* and *crzA* genes rendered *A. niger* moderately sensitive to CA and AbaA. It thus becomes clear that the contribution of MsnA and CrzA to cell wall strengthening is only of limited importance (Figure 6). (v) RlmA, MsnA and CrzA do not function in the protection of *A. niger* against the calcium signaling inhibitor FK506, as the respective single or double deletion strains do not show altered susceptibilities against this calcineurin inhibitor. (vi) Similarly, the function of RlmA and MsnA is not important to counteract the growth inhibitory effects of FP; rather, the transcriptomic fingerprint proposes that the transcription factor SrbA is a likely regulator for this survival response.

## Conclusions

The present work uncovered different defence strategies of *A. niger* to protect itself against cell wall stress conditions. At least three transcription factors - RlmA, MsnA and CrzA – are employed in an obvious sophisticated and well-balanced manner. The data also predicts a fourth factor, SrbA, which seems to be specifically important during cell membrane stress. Future studies will disclose how these regulators are interlocked in different signaling pathways to secure survival under different stress conditions.

## Methods

### Strains, growth conditions and antifungal compounds

*A. niger* strains used in this study are listed in Table 3. The strains were grown at 30°C (unless otherwise stated) in minimal medium (MM) [61] or complete medium (CM), consisting of minimal medium (MM) supplemented with 1% yeast extract and 0.5% casamino acids. Fermentation medium (FM) was composed of 0.75% glucose, 0.45% NH_4_Cl, 0.15% KH_2_PO_4_, 0.05% KCl, 0.05% MgSO_4_, 0.1% salt solution [61] and 0.003% yeast extract. The pH of FM was adjusted to pH 3. Aureobasidin A was purchased from Takara Bio, FK506 from A.G. Scientific, fenpropimorph from Sigma Aldrich, caspofungin (Cancidas®) from Merck and calcofluor white from BASF.

**Table 3:** ***Aspergillus niger*** **strains used in this work**

Name	Genotype	Reference
N402	*cspA1, amdS* ^*−*^	[62]
MA169.4	*kusA*::DR*-amdS-* DR*, pyrG*^*−*^	[63]
MA78.6	*ΔkusA, pyrG*^*+*^(derivative of MA70.15 containing *A. niger pyrG*)	[63]
MA47.1	*ΔkusA, pyrG*^*+*^, *ΔrlmA (* derivative of AB4.1)	[12]
ER2.5	*ΔkusA, ΔrhoB::AopyrG*	[13]
ER3.4	*ΔkusA, ΔrhoC::AopyrG*	[13]
ER7.6	*ΔkusA, ΔrhoD::AopyrG*	[13]
MA84.1	*ΔkusA, ΔcftA::hygR*	[13]
MA234.1	*pyrG*^*+*^ (derivative of N402 containing *A. niger pyrG*)	Unpublished
8.21	*ΔcrzA, pyrG*^*+*^(derivative of MA234.1)	Unpublished
MF3.2	*kusA*::DR*-amdS-* DR*, ΔrlmA, hph*	This study
JH1.1	*kusA::DR-amdS-DR, msnA* ^*−*^ *, hph*	This study
MF4.10	*kusA*::DR*-amdS-* DR*, msnA*^*−*^ *, ΔrlmA, hph*	This study

### Screening for antifungal-induced morphological changes

5 × 10^5^ conidia of an *A. niger* strain were inoculated in Petri dishes containing 5 ml of liquid MM supplemented with 0.003% yeast extract. Prior to inoculation, coverslips were placed onto the bottom of the Petri dishes. Spores were allowed to germinate for 5 h at 37°C until small germ tubes became visible in more than 90% of the spores. Compounds were added at various concentrations. The negative control was supplemented with the same volume of solvent (ethanol or DMSO). After further cultivation for 1 h at 37°C, germlings that were adherent to the coverslips were analyzed by microscopy (see below). From at least 100 germlings per sample, the morphology was characterized as being either unbranched (germlings with a single germ tube) or branched (germlings with apical and/or subapical branches). The Branching Index was defined as BI = (Σ branched germlings) × (Σ branched + unbranched germlings)^−1^.

### Growth assays in microtiter plates

10^4^ conidia of an *A. niger* strain were inoculated in each well of 96-well optical glass bottom microtiter plates (Nunc art) in 200 μl MM supplemented with 0.003% yeast extract and cultivated for 30 hours at 30°C. Different concentrations of antifungals were supplemented prior to inoculation, whereby the negative controls were supplemented with the same volume of solvent (H_2_O, ethanol or DMSO). The effect of each compound was tested for at least 3 different concentrations in triplicates and each experiment was performed at least twice. Biomass accumulation was measured at fixed intervals at OD_590_.

### Growth-plate inhibition assays

Defined spore titers of *A. niger* strains were used to inoculate MM plates supplemented with different concentrations of stress agents and incubated for 1–3 days at 30, 37 and 42°C, respectively. All experiments were performed at least in duplicates.

### Bioreactor cultivation

Freshly harvested conidia (5 × 10^8^) from strain N402 were used to inoculate 0.5 liters of FM. Cultivations were performed in BioFlo/CelliGen 115 bioreactors (New Brunswick Scientific) as described earlier [14]. In brief, 250 rpm was used as agitation speed and aeration was performed via the headspace until the dissolved oxygen tension dropped to 40%. Thereafter, aeration was switched to sparger aeration. Temperature and pH were set to 30°C and pH 3, respectively, and controlled on-line using the program NBS Biocommand. After 5 h of cultivation, AbaA (dissolved in 5 ml ethanol) or FK506 (dissolved in 5 ml DMSO) was added. 5 ml of ethanol or 5 ml of DMSO were added in the control runs. After an additional hour of cultivation, 500 ml of the culture broth were quickly harvested via filtration, and mycelial samples were immediately frozen using liquid nitrogen. In addition, samples were taken for microscopic analysis (see below) and calculation of the BI value. Note that the *ΔrlmA* deletion strain was cultivated 6 h instead of 5 h, as described previously [14].

### Microscopy

Pictures of *A. niger* germlings were captured using an Axioplan 2 (Zeiss) equipped with a DKC-5000 digital camera (Sony). Light (using DIC settings) images were obtained with a 40× objective. Images were processed using Adobe Photoshop 6.0 (Adobe Systems Inc.).

### RNA extraction, expression profiling

Total RNA isolation, RNA quality control, labeling, Affymetrix chip hybridization, scanning and signal calculation were performed as described previously [14]. Microarray analyses were performed for biological duplicates for each condition (controls, FK506-, and AbaA-treated samples). Expression data was analyzed using the open source programs R (http://www.r-project.org/) and Bioconductor (http://www.bioconductor.org/). Background correction, normalization and probe summarization was performed using the default setting of the robust multi-array analysis (RMA) package as recently described [64]. Differential gene expression was evaluated by moderated t-statistics using the Limma package [65] with a threshold of the Benjamini and Hochberg False Discovery Rate (FDR) of 0.05 [66]. Fold change of gene expression from different samples was calculated from normalized expression values. Geometric means of the expression values as well as fold change for all conditions and comparisons are summarized in Additional file 1 and Additional file 2 and have been deposited at the GEO repository (http://www.ncbi.nlm.nih.gov/geo/) under the accession number GSE56471.

### Bioinformatics

Responsive genes in the antifungal-treated samples were functionally classified into FunCat categories as described previously [14],[19]. *In silico* analysis of putative transcription factor binding sites localized in the 1,000-bp upstream regions the responsive genes was performed using the transcription factor binding site finder (TFBSF) tool [48]. In brief, the upstream regions of the differentially expressed genes were searched for the presence of putative binding sites recognized by 25 known transcription factors from *Aspergillus* or *Trichoderma* species (see Additional file 9). To determine significant over- or underrepresentation of binding sites, the background distribution of the identified motifs in the genome of *A. niger* was determined via bootstrapping (500,000 bootstraps).

### Inactivation of *rlmA* and *msnA* genes in *A. niger*

To inactivate the *rlmA* gene, a deletion approach was followed as described previously [12]. Construct pΔRlmA [12] was used to delete the *rlmA* gene of *A. niger* in the *ΔkusA* background strain MA169.4 [63]. The plasmid was linearized with BglI prior to transformation. Transformants were purified twice on MM plates lacking uridine to obtain homokaryotic mycelium (*pyrG*^+^). Successful deletion was verified via Southern analysis (Additional file 10). Strain MF3.2 was selected for further analyses.

To inactivate the *msnA* gene, a disruption approach was followed. A 659 bp long fragment of the *msnA* gene comprising part of its 5’ open reading frame was amplified using the primers MsnA_fw_hind and MsnA_rev_hind (Additional file 11) and cloned into the unique restriction site HindIII of pAN7.1 [67]. Using the *hph* gene for hygromycin B resistance as a selective marker, the resulting vector pJH1.56 was co-transformed together with pAB4.1 [68] into MA169.4. This co-transformation approach was necessary to change the *pyrG*^−^ genetic background of MA169.4 into *pyrG*^+^. Transformants were purified twice on MM plates containing 100 μg/ ml hygromycin B and lacking uridine to obtain homokaryotic mycelium (hygB, *pyrG*^+^). Correct disruption of the *msnA* disruption cassette was verified via Southern analysis (Additional file 10). Strain JH1.1 was selected for further analyses.

To obtain a strain in which both *rlmA* and *msnA* genes were inactivated, strain MF3.2 (*ΔrlmA*) was transformed with the *msnA* disruption construct pJH1.56. Transformants were purified twice on MM containing 100 μg/ ml hygromycin B. Correct disruption of the *msnA* disruption cassette was verified via Southern analysis (Additional file 10). Strain MF4.10 was selected for further analyses.

## Availability of supporting data

The data sets supporting the results of this article are available in the GEO repository, (http://www.ncbi.nlm.nih.gov/geo/) under the accession number GSE56471.

## Additional files

## Electronic supplementary material

Additional file 1: Table S1.: Expression data. (XLSX 10 MB)

Additional file 2: Table S2.: Gene expression comparison. (XLSX 552 KB)

Additional file 3: Table S3.: Selected aureobasidin A responsive genes ordered into different biological processes. (DOCX 36 KB)

Additional file 4: Table S4.: Selected FK506 responsive genes ordered into different biological processes. (DOCX 34 KB)

Additional file 5: Table S5.: Selected caspofungin responsive genes ordered into different biological processes. (DOCX 35 KB)

Additional file 6: Table S6.: Selected fenpropimorph responsive genes ordered into different biological processes. (DOCX 30 KB)

Additional file 7: Table S7.: Selected genes responsive to the deletion of RlmA ordered into different biological processes. (DOCX 34 KB)

Additional file 8: Table S8.: List of overlapping genes from Venn Diagram analysis. (XLS 370 KB)

Additional file 9: Table S9.: Results of the TFBSF for the caspofungin dataset. (XLS 46 KB)

Additional file 10: Figure S1.: Southern blot of MF3.2, JH1.1 and MF4.10. (PNG 1 MB)

Additional file 11: Table S10.: Primers Used in this study. (XLS 34 KB)

Below are the links to the authors’ original submitted files for images.

Authors’ original file for figure 1

Authors’ original file for figure 2

Authors’ original file for figure 3

Authors’ original file for figure 4

Authors’ original file for figure 5

Authors’ original file for figure 6

Authors’ original file for figure 7

Authors’ original file for figure 8

Authors’ original file for figure 9

Authors’ original file for figure 10
