# Supplementary material for: The capacity of Aspergillus niger to sense and respond to cell wall stress requires at least three transcription factors: RlmA, MsnA and CrzA
Source: Fungal Biol Biotechnol. 2014 Dec 1;1:5. doi: 10.1186/s40694-014-0005-8 (PMC5598236; doi:10.1186/s40694-014-0005-8)
Supplement: Supplementary file 3 — Additional file 3: Table S3.: Selected aureobasidin A responsive genes ordered into different biological processes. (DOCX 36 KB) [file 40694_2014_5_MOESM3_ESM.docx]

**SUPPLEMENTAL TABLE S5:** Selected aureobasidin A responsive genes ordered into different biological processes

| ORF code | Gene | Up/Down | (Predicted) protein function | Closest  *S. cerevisiae* ortholog |
| --- | --- | --- | --- | --- |
| **Cell wall synthesis and integrity** | | | |  |
| An14g05530 | *rhoD* | ↑ | Rho GTPase | Rho4 |
| An14g00660 | *chsA* | ↑ | chitin synthase |  |
| An12g10380 | *chsF* | ↑ | chitin synthase |  |
| An04g04670 | *chiB* | ↑ | chitinase | Cts2 |
| An15g07810 | *agsB* | ↓ | α-1,3-glucan synthase |  |
| An01g03090 | *engA* | ↑ | glucan endo-1,3-beta-D-glucosidase | Dse4 |
| An04g07150 |  | ↑ | protein involved in cell wall biogenesis | Ecm15 |
| An01g04650 |  | ↑ | nucleoside diphosphate-sugar epimerase |  |
| An04g04630 |  | ↑ | capsule protein |  |
| **Cytoskeleton** |  |  |  |  |
| An02g06360 | *arc16* | ↑ | subunit of Arp2/3 complex |  |
| An05g00810 | *tbcA* | ↑ | tubulin specific chaperone |  |
| An11g03390 |  | ↑ | actin binding protein | Ysc84 |
| **Lipid metabolism** | | | |  |
| An16g07110 | *ach1* | ↑ | acetyl-CoA hydrolase |  |
| An02g01180 | *dppA* | ↑ | diacylglycerol pyrophosphate phosphatase, synthesis of DAG | Dpp1 |
| An05g01210 |  | ↑ | 1-acyldihydroxyacetone-phosphate reductase |  |
| An05g01070 |  | ↑ | LCB transporter | Rsb1 |
| An02g02820 | *echA* | ↑ | enoyl-CoA hydratase |  |
| An17g01150 | *acdA* | ↑ | acyl-CoA dehydrogenase |  |
| An04g00040 |  | ↑ | malonyl transferase |  |
| An18g01590 | *acuJ* | ↑ | carnitine acetyltransferase |  |
| An08g10110 |  | ↑ | lipid transfer protein |  |
| An15g00630 | *mitA* | ↑ | MIPC synthase |  |
| An08g04090 |  | ↑ | acetoacetat-CoA ligase |  |
| An04g01370 | *fatD* | ↑ | malonyl CoA synthetase |  |
| An07g00440 |  | ↑ | triacylglycerol lipase |  |
| An03g03360 | *acuH* | ↑ | carnitine transporter |  |
| An18g05210 |  | ↑ | peroxisomal dehydratase |  |
| An01g00120 |  | ↑ | enoyl-[acyl-carrier-protein] reductase |  |
| **Vesicle transport** | |  |  |  |
| An14g00010 | *srgA* | ↑ | Rab GTPase | Sec4 |
| An01g03770 | *nudG* | ↑ | dynein light chain |  |
| An02g12980 | *sed5* | ↑ | t-SNARE required for vesicle transport between ER and Golgi | Sed5p |
| An18g06500 | *sec53* | ↑ | phosphomannomutase |  |
| An07g05990 |  | ↑ | vacuolar -SNARE with phosphatidylinositol binding activity |  |
| An13g01040 |  | ↑ | Rab geranylgeranyl transferase | Bet2 |
| **Transporter** | |  |  |  |
| An06g02270 |  | ↑ | arabinose transporter |  |
| An12g07450 | *mstA* | ↑ | sugar symporter |  |
| An01g08780 |  | ↑ | hexose transporter |  |
| An07g04430 |  | ↑ | hexose transporter |  |
| An01g00850 | *xylT* | ↑ | xylose permease |  |
| An04g00340 |  | ↑ | myo-inositol transport protein |  |
| An06g01900 |  | ↑ | phosphatidylinositol transporter |  |
| An08g02290 |  | ↓ | polyamine transporter |  |
| **Carbohydrate metabolism** | |  |  |  |
| An02g08000 | *ganA* | ↑ | G-protein alpha subunit, activator of cAMP signaling | Gpa2 |
| An01g10930 | *agdB* | ↑ | α-glucosidase |  |
| An01g09270 | *acuD* | ↑ | isocitrate lyase |  |
| An03g03740 | *bgl4* | ↑ | β-glucosidase |  |
| An07g08390 |  | ↑ | oxaloacetate acetyl hydrolase |  |
| An02g04900 | *pgaB* | ↑ | endopolygalacturonase |  |
| An18g03570 | *bglA* | ↑ | β-glucosidase |  |
| An01g01540 | *treA* | ↑ | α,α-trehalase |  |
| An16g04770 |  | ↑ | aldo/keto reductase |  |
| An04g05620 | *acuA* | ↑ | acetate-CoA ligase |  |
| An12g00030 | *xdhA* | ↑ | D-xylulose reductase |  |
| **Proteasome** |  |  |  |  |
| An04g01870 | *pre1* | ↑ | subunit of the 20S proteasome |  |
| An07g07240 |  | ↑ | E3 ubiquitin ligase |  |
| An14g00180 |  | ↑ | subunit of the 19S proteasome regulatory particle |  |
| An18g06700 | *pre7* | ↑ | subunit of the 20S proteasome 20S |  |
| An13g01210 |  | ↑ | subunit of the 20S proteasome |  |
| An18g06680 | *pre4* | ↑ | subunit of the 20S proteasome |  |
| An02g07040 | *scl1* | ↑ | subunit of the 20S proteasome |  |
| An14g03930 |  | ↑ | maturation factor of the 20 S proteasome |  |
| An03g04140 |  | ↑ | N-terminal asparagine amidohydrolase |  |
| **Transcription factor** | |  |  |  |
| An12g03170 |  | ↑ | transcription factor | Cha4 |
| An07g07050 |  | ↑ | transcription factor | Asg1 |
| An07g05960 |  | ↑ | transcription factor | Msn2/4 |
| An15g02080 |  | ↑ | transcription factor | Hap1 |
| An12g09020 |  | ↑ | transcription factor | Hal9 |

Genes up-regulated are indicated with ↑, genes down-regulated with ↓. Differential gene expression was evaluated by moderated t-statistics using the Limma package [63] with a FDR threshold at 0.05 [64]. *: Protein functions were predicted based on information inferred from the *Saccharomyces* genome data base SGD (http://www.yeastgenome.org/) and the *Aspergillus* genome database AspGD (http://www.aspergillusgenome.org/).
