# Supplementary material for: The capacity of Aspergillus niger to sense and respond to cell wall stress requires at least three transcription factors: RlmA, MsnA and CrzA
Source: Fungal Biol Biotechnol. 2014 Dec 1;1:5. doi: 10.1186/s40694-014-0005-8 (PMC5598236; doi:10.1186/s40694-014-0005-8)
Supplement: Supplementary file 4 — Additional file 4: Table S4.: Selected FK506 responsive genes ordered into different biological processes. (DOCX 34 KB) [file 40694_2014_5_MOESM4_ESM.docx]

**SUPPLEMENTAL TABLE S6:** Selected FK506 responsive genes ordered into different biological processes

| ORF code | Gene | Up/down | (Predicted) protein function | Closest  *S. cerevisiae*  ortholog |
| --- | --- | --- | --- | --- |
| **Calcium signaling** | |  |  |  |
| An19g00340 |  | ↑ | vacuolar H^+^/Ca^2+^ antiporter | Vcx1 |
| An19g00330 |  | ↑ | vacuolar H^+^/Ca^2+^ antiporter | Vcx1 |
| An19g00320 |  | ↑ | vacuolar H^+^/Ca^2+^ antiporter | Vcx1 |
| An19g00350 |  | ↑ | calcium-transporting ATPase | Pmc1 |
| **Cell surface signaling** | | | | |
| An04g02930 |  | ↑ | G-protein-coupled receptor similar to *Magnaporthe grisea* Pth11 involved in appressorium formation | / |
| An02g01560 | *gprD* | ↓ | G-protein coupled receptor for lysophosphatidic acid | / |
| **Transporter** |  |  |  |  |
| An01g01950 |  | ↑ | plasma membrane Mg^2+^ transporter | Alr2 |
| An05g00640 |  | ↓ | plasma membrane Mg^2+^ transporter | Alr2 |
| An02g01480 |  | ↓ | multidrug efflux transporter | Yhk8 |
| An14g06950 |  | ↓ | multidrug efflux transporter | Tpo2 |
| An12g10320 | *zrtA* | ↑ | zinc transporter | Zrt1 |
| An07g06300 |  | ↑ | hexose transporter | Hxt16 |
| An16g06580 |  | ↑ | quinate transporter | Hxt2 |
| **Lipid metabolism** | |  |  |  |
| An02g13220 | *lplB* | ↑ | lysophospholipase phospholipase B | Plb1 |
| An16g07890 |  | ↓ | transcription factor involved in sterol metabolism | Ecm22 |
| **Vesicle transport and protein secretion** | | | |  |
| An15g03330* |  | ↑ | Golgi α-1,6-mannosyltransferase | Mnn10 |
| An09g05420* |  | ↑ | subunit of the signal peptidase complex | Spc3 |
| An03g04940* |  | ↑ | protein localized to COPII-coated vesicles | Erv41 |
| An01g08420* | *clxA* | ↑ | ER chaperone calnexin | Cnx1 |
| An01g04600* | *prpA* | ↑ | protein disulfide isomerase | Mpd1 |
| An03g04410* |  | ↑ | UDP-glucose:dolichyl-phosphate glucosyltransferase | Alg5 |
| An02g01510* |  | ↑ | subunit of SEC63 complex | Sec62 |
| An02g14930* |  | ↑ | subunit of the OST complex of the ER lumen | Ost3 |
| An15g07200 |  | ↑ | synaptotagmin C2 domain-containing protein |  |
| An16g06820 |  | ↑ | ER curvature-stabilizing protein | Yop1 |
| An15g07160 | *pelF* | ↑ | pectin lyase |  |
| An08g00740* |  | ↓ | subunit of the endosomal sorting complex ESCRT III | Vps20 |
| **Transcription factor** | |  |  |  |
| An16g08130 |  | ↑ | transcription factor involved in acetamide catabolism, similar to AmdX in *A. nidulans* | Tda9 |
| An15g02080 |  | ↓ | transcription factor | Hap1 |

Genes up-regulated are indicated with ↑, genes down-regulated with ↓. Differential gene expression was evaluated by moderated t-statistics using the Limma package [63] with a FDR threshold at 0.05 [64]. *: Protein functions were predicted based on information inferred from the *Saccharomyces* genome data base SGD (http://www.yeastgenome.org/) and the *Aspergillus* genome database AspGD (<http://www.aspergillusgenome.org/>). /: *S. cerevisiae* does not contain an orthologous protein. ORFs labeled with * also respond to the ER-stress agents tunicamycin or DTT [36].
