# Supplementary material for: The capacity of Aspergillus niger to sense and respond to cell wall stress requires at least three transcription factors: RlmA, MsnA and CrzA
Source: Fungal Biol Biotechnol. 2014 Dec 1;1:5. doi: 10.1186/s40694-014-0005-8 (PMC5598236; doi:10.1186/s40694-014-0005-8)
Supplement: Supplementary file 5 — Additional file 5: Table S5.: Selected caspofungin responsive genes ordered into different biological processes. (DOCX 35 KB) [file 40694_2014_5_MOESM5_ESM.docx]

**SUPPLEMENTAL TABLE S7:** Selected caspofungin responsive genes ordered into different biological processes

| ORF code | Gene | Up/down | (Predicted) protein function | Closest  *S. cerevisiae* ortholog |
| --- | --- | --- | --- | --- |
| **Cell wall synthesis and CWI signaling** | | | | |
| An16g04200 | *rhoB* | ↑ | Rho GTPase | Rho2 |
| An10g00490 | *rapA* | ↑ | Rho-GAP | Sac7 |
| An18g04590 |  | ↑ | Rho-GDP dissociation inhibitor | Rdi1 |
| An18g03740 | *mkkA* | ↑ | MAP kinase kinase | Mkk2 |
| An14g01820 | *phiA* | ↑ | cell wall protein | / |
| An12g10200 |  | ↑ | cell wall protein | / |
| An03g05940 | *gfaB* | ↑ | glutamine:fructose-6-phosphate amidotransferase | Gfa1 |
| An04g09890 | *agsA* | ↑ | cell wall alpha-glucan synthase | / |
| An09g04010 | *chsB* | ↑ | chitin synthase | Chs1 |
| An12g07840 | *gnaA* | ↑ | glucosamine 6-phosphate N-acetyltransferase | Gna1 |
| An10g00400 | *gelA* | ↑ | 1,3-beta-glucanosyltransferase | Gas5 |
| An07g07530 | *crhB* | ↑ | GPI-anchored glucanosyltransferase | Utr2 |
| An18g06820 | *gfaA* | ↑ | glutamine:fructose-6-phosphate amidotransferase | Gfa1 |
| An16g02850 | *crhD* | ↑ | transglycosidase | Crh1 |
| An09g03070 | *agsE* | ↑ | ɑ-1,3-glucan synthase | / |
| An01g04650 |  | ↑ | nucleoside diphosphate-sugar epimerase |  |
| An02g07650 | *pgmB* | ↑ | phosphoglucomutase | Pgm2 |
| An07g05820 |  | ↑ | transmembrane protein |  |
| An02g09550 | [*hetC2*](http://www.aspergillusgenome.org/cgi-bin/locus.pl?locus=An02g09550&organism=A_niger_CBS_513_88#ASPL0000268629) | ↑ | glycolipid transfer protein |  |
| An14g03520 | *dfgC* | ↑ | GPI-anchored endomannanase | Dcw1 |
| An02g02340 | *csmB* | ↑ | chitin synthase with a myosin motor-like domain | Chs3 |
| An12g00480 |  | ↑ | UDP-N-acetylglucosamine pyrophosphorylase | Qri1 |
| An02g07590 |  | ↑ | N-acetyltransferase |  |
| An02g14500 |  | ↑ | GPI-anchored cell wall protein |  |
| **Secretion** |  |  |  |  |
| An14g00010 | *srgA* | ↑ | Rab related GTPase | Sec4 |
| An09g06790 | *srgB* | ↑ | Rab related GTPase | Ypt1 |
| An04g02020 | *cypB* | ↑ | ER peptidyl-prolyl cis-trans isomerase | Cpr1 |
| An03g04410 |  | ↑ | UDP-glucose:dolichyl-phosphate glucosyltransferase | Alg5 |
| An13g00620 |  | ↑ | glucosidase II, beta subunit | Gtb1 |
| An05g00140 |  | ↑ | beta subunit of signal recognition particle receptor | Srp102 |
| An07g05800 |  | ↑ | signal recognition particle protein | Srp14 |
| An18g02020 | *tigA* | ↑ | protein disulfide isomerase | Pdi1 |
| An01g06670 |  | ↑ | peptidyl-prolyl isomerase, FK506 binding | Fpr2 |
| An01g00560 |  | ↑ | signal peptidase complex subunit | Sec11 |
| An04g07040 |  | ↑ | clathrin light chain | Clc1 |
| An16g04330 | *dpmA* | ↑ | ER dolichyl-phosphate beta-d-mannosyltransferase | Dpm1 |
| An18g06500 |  | ↑ | phosphomannomutase | Sec63 |
| An01g11630 | *sec61A* | ↑ | subunit of the SEC61 complex | Sss1 |
| An13g01040 |  | ↑ | geranylgeranyltransferase | Bet2 |
| An12g00380 |  | ↑ | component of the TRAPP complex | Bet3 |
| An08g03590 |  | ↑ | component of COPII-coated vesicles | Emp24 |
| **Cytoskeleton** |  |  |  |  |
| An08g06410 |  | ↑ | component of the Arp2/3 complex | Arp2 |
| An01g05510 |  | ↑ | subunit of the Arp2/3 complex | Arc35 |
| An18g06590 |  | ↑ | subunit of the Arp2/3 complex | Arc40 |
| An13g00760 | *tpm1* | ↑ | tropomyosin | Tpm1 |
| An01g03770 |  | ↑ | dynein light chain | Dyn2 |
| An05g00810 | *tbcA* | ↑ | tubulin-specific chaperone | Rbl2 |
| An18g03900 |  | ↑ | prefoldin subunit 2 involved in actin/tubilin folding | Gim4 |
| **Lipid metabolism** | |  |  |  |
| An08g10110 |  | ↑ | lipid transfer protein |  |
| An06g01900 |  | ↑ | Phosphatidylinositol transfer protein | Csr1 |
| **Proteasome** |  |  |  |  |
| An18g06700 |  | ↑ | 20S proteasome core subunit | Pre7 |
| An14g00180 |  | ↑ | 19S proteasome regulatory particle subunit | Rpt6 |
| An18g06680 |  | ↑ | 20S proteasome core subunit | Pre4 |
| An04g01870 |  | ↑ | 20S proteasome core subunit | Pre1 |
| **Vacuolar integrity** | |  |  |  |
| An12g08760 |  | ↑ | vacuolar ATPase subunit E | Vma4 |
| An12g00640 |  | ↑ | vacuolar ATPase subunit G | Vma10 |
| An02g03300 |  | ↑ | vacuolar ATPase subunit F | Vma7 |
| An15g06910 |  | ↑ | Palmitoyltransferase for Vac8 | Pfa3 |

Genes up-regulated are indicated with ↑, genes down-regulated with ↓. Differential gene expression was evaluated by moderated t-statistics using the Limma package [63] with a FDR threshold at 0.05 [64]. *: Protein functions were predicted based on information inferred from the *Saccharomyces* genome data base SGD (http://www.yeastgenome.org/) and the *Aspergillus* genome database AspGD (<http://www.aspergillusgenome.org/>). /: *S. cerevisiae* does not contain an orthologous protein.
