# Supplementary material for: The capacity of Aspergillus niger to sense and respond to cell wall stress requires at least three transcription factors: RlmA, MsnA and CrzA
Source: Fungal Biol Biotechnol. 2014 Dec 1;1:5. doi: 10.1186/s40694-014-0005-8 (PMC5598236; doi:10.1186/s40694-014-0005-8)
Supplement: Supplementary file 6 — Additional file 6: Table S6.: Selected fenpropimorph responsive genes ordered into different biological processes. (DOCX 30 KB) [file 40694_2014_5_MOESM6_ESM.docx]

**SUPPLEMENTAL TABLE S8:** Selected fenpropimorph responsive genes ordered into different biological processes

| ORF code | Gene | Up/down | (Predicted) protein function | Closest |
| --- | --- | --- | --- | --- |
|  |  |  |  | *S. cerevisiae* ortholog |
| **Lipid metabolism** | | | | |
| An08g05400 |  | ↑ | acetyl-CoA acetyltransferase | Pot1 |
| An17g01150 |  | ↑ | acyl-CoA dehydrogenase |  |
| An15g01280 |  | ↑ | peroxisomal Δ3,Δ2-enoyl-CoA isomerase | Ecl1 |
| An16g04520 |  | ↑ | 3-oxoacyl-(acyl carrier protein) reductase | Fox2 |
| An08g07520 |  | ↑ | 3-oxoacyl-(acyl-carrier-protein) reductase |  |
| An14g00990 |  | ↑ | Enoyl-CoA hydratase | Fox2 |
| An16g05340 |  | ↑ | enoyl-ACP reductase |  |
| An07g03290 |  | ↑ | enoyl-ACP reductase |  |
| An15g02830 |  | ↑ | mitochondrial acetyl-coenzyme A carboxylase, putative | Hfa1 |
| An03g05170 | *srbA* | ↑ | transcription factor | Hms1 |
| An01g03350 |  | ↑ | C-8 sterol isomerase | Erg2 |
| An15g02820 |  | ↑ | pyruvate carboxylase | Pyc2 |
| An18g01590 |  | ↑ | mitochondrial carnitine acety-CoA ltransferase | Cat2 |
| An04g00740 |  | ↑ | sterol carrier protein |  |

Genes up-regulated are indicated with ↑, genes down-regulated with ↓. Differential gene expression was evaluated by moderated t-statistics using the Limma package [63] with a FDR threshold at 0.05 [64]. *: Protein functions were predicted based on information inferred from the *Saccharomyces* genome data base SGD (http://www.yeastgenome.org/) and the *Aspergillus* genome database AspGD (http://www.aspergillusgenome.org/).
