# Supplementary material for: The capacity of Aspergillus niger to sense and respond to cell wall stress requires at least three transcription factors: RlmA, MsnA and CrzA
Source: Fungal Biol Biotechnol. 2014 Dec 1;1:5. doi: 10.1186/s40694-014-0005-8 (PMC5598236; doi:10.1186/s40694-014-0005-8)
Supplement: Supplementary file 7 — Additional file 7: Table S7.: Selected genes responsive to the deletion of RlmA ordered into different biological processes. (DOCX 34 KB) [file 40694_2014_5_MOESM7_ESM.docx]

**SUPPLEMENTAL TABLE S9:** Selected genes responsive to the deletion of RlmA ordered into different biological processes

| ORF code | Gene | Up/down | (Predicted) protein function | Closest  *S. cerevisiae* ortholog | |
| --- | --- | --- | --- | --- | --- |
| **Cell wall and membrane remodeling** | | |  |  | |
| An11g01240 | *dfgB* | ↑ | GPI-anchored mannosidase | Dcw1 | |
| An18g06500 |  | ↑ | phosphomannomutase | Sec53 | |
| An15g01420 |  | ↑ | alpha glucosidase I for ɑ-1,6-glucan synthesis | Cwh41 | |
| An09g03070 | *agsE* | ↓ | ɑ-1,3-glucan synthase | / | |
| **Secretion** | |  |  |  | |
| An14g00010 | *srgA* | ↑ | Rab-related GTPase | Sec4 | |
| An09g06790 | *srgB* | ↑ | Rab-related GTPases | Ypt1 | |
| An14g00850 |  | ↑ | peptidyl-prolyl isomerase, negative regulator of calcineurin and TOR signaling | Fpr1 | |
| An12g00380 |  | ↑ | Component of the TRAPP complex | Bet3 | |
| An04g02020 | *cypB* | ↑ | ER peptidyl-prolyl cis-trans isomerase | Cpr1 | |
| An18g05830 | *kapI* | ↓ | Protein secretion enhancer | Pse1 | |
| An15g01380 |  | ↑ | v-SNARE | Sec22 | |
| An16g04330 | *dpmA* | ↑ | ER dolichyl-phosphate beta-d-mannosyltransferase | Dpm1 | |
| An05g00140 |  | ↑ | beta subunit of signal recognition particle receptor | Srp102 | |
| An08g00300 |  | ↑ | guanylate kinase required for mannose elongation of N-linked glycoproteins | Guk1 | |
| An01g11630 | *sec61A* | ↑ | subunit of the SEC61 complex | Sss1 | |
| An04g05980 |  | ↑ | retrograde v-SNARE | Vti1 | |
| An14g04880 |  | ↑ | component of the ESCRT III complex | Did4 | |
| An13g00620 |  | ↑ | beta subunit of glucosidase II | Gtb1 | |
| An01g05200 |  | ↑ | dolichol-phosphate mannosyltransferase subunit 2 |  | |
| An07g05800 |  | ↑ | signal recognition particle protein | Srp14 | |
| An18g02020 | *tigA* | ↑ | protein disulfide isomerase | Pdi1 | |
| An03g04410 |  | ↑ | dolichyl-phosphate glucosyltransferase | Alg5 | |
| An02g01580 |  | ↑ | vesicular transport protein | Sec17 | |
| An03g06550 | *glaA* | ↑ | glucoamylase |  | |
| An18g03920 |  | ↑ | oligosaccharyltransferase complex | Ost2 | |
| **Cytoskeleton** | |  |  | |  |
| An05g00810 | *tbcA* | ↑ | tubulin-specific chaperone | Rbl2 | |
| An01g05510 |  | ↑ | subunit of the Arp2/3 complex | Arc35 | |
| An18g06590 |  | ↑ | subunit of the Arp2/3 complex | Arc40 | |
| An13g00760 | *tpmA* | ↑ | tropomyosin | Tpm1 | |
| An01g05290 |  | ↑ | beta subunit of the capping protein heterodimer | Cap2 | |
| **Proteasome** | |  |  |  | |
| An04g06510 |  | ↑ | ubiquitin-like protein | Ubi4 | |
| An17g00260 |  | ↑ | ubiquitin protein ligase | Ubc1 | |
| An09g04000 |  | ↑ | E2 ubiquitin-conjugating enzyme | Ubc12 | |
| An18g06800 |  | ↑ | subunit of the 20S proteasome | Pre10 | |
| An18g06700 |  | ↑ | subunit of the 20S proteasome | Pre7 | |
| An13g01210 |  | ↑ | subunit of the 20S proteasome | Pre3 | |
| An18g06680 |  | ↑ | subunit of the 20S proteasome | Pre4 | |
| An02g07040 |  | ↑ | subunit of the 20S proteasome | Scl1 | |
| An14g00180 |  | ↑ | 19S proteasome regulatory particle | Rpt6 | |
| An08g02790 |  | ↑ | 19S proteasome regulatory particle | Nas6 | |
| An08g10710 |  | ↑ | 19S proteasome regulatory particle | Rpn9 | |
| An07g09730 |  | ↓ | ubiquitin-specific protease | Ubp3 | |
| An11g01610 |  | ↓ | ubiquitin-protein ligase | Ubr1 | |
| An02g01420 |  | ↓ | ubiquitin carboxyl-terminal hydrolase | Ubp12 | |
| **Vacuolar integrity** | | | | | |
| An18g02210 |  | ↑ | Rab-related GTPase | Ypt7 | |
| An02g03300 |  | ↑ | vacuolar ATPase subunit F | Vma7 | |
| An07g05080 |  | ↑ | vacuolar ATPase subunit C | Vma11 | |
| An02g09250 | *vmaD* | ↑ | vacuolar ATPase subunit D | Vma6 | |

Genes up-regulated are indicated with ↑, genes down-regulated with ↓. Differential gene expression was evaluated by moderated t-statistics using the Limma package [63] with a FDR threshold at 0.05 [64]. *: Protein functions were predicted based on information inferred from the *Saccharomyces* genome data base SGD (http://www.yeastgenome.org/) and the *Aspergillus* genome database AspGD (http://www.aspergillusgenome.org/). /: *S. cerevisiae* does not contain an orthologous protein.
