# Supplementary figures and images for: The capacity of Aspergillus niger to sense and respond to cell wall stress requires at least three transcription factors: RlmA, MsnA and CrzA
Source: Fungal Biol Biotechnol. 2014 Dec 1;1:5. doi: 10.1186/s40694-014-0005-8 (PMC5598236; doi:10.1186/s40694-014-0005-8)

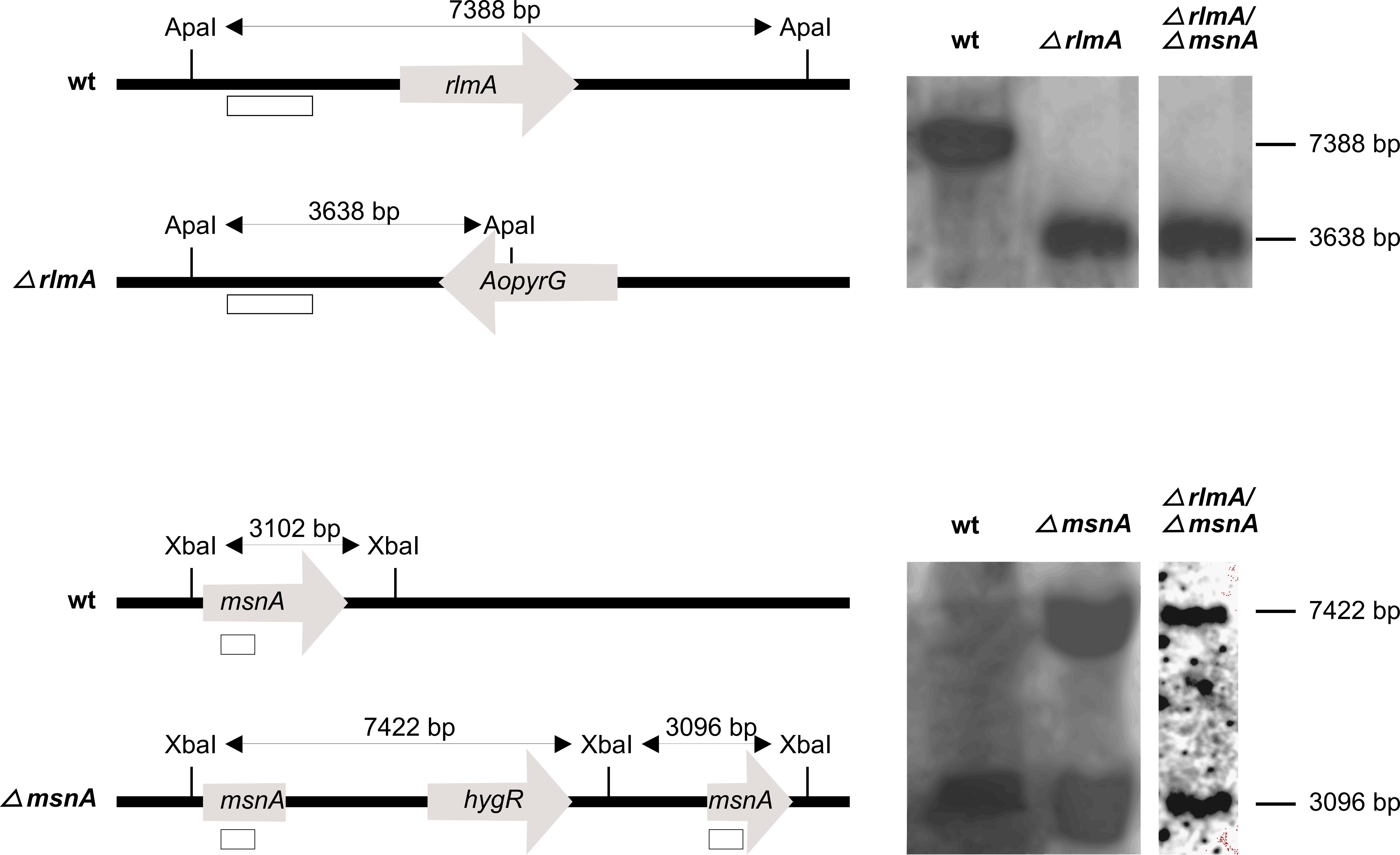

Supplement: Supplementary file 10 — Additional file 10: Figure S1.: Southern blot of MF3.2, JH1.1 and MF4.10. (PNG 1 MB) [file 40694_2014_5_MOESM10_ESM.png]

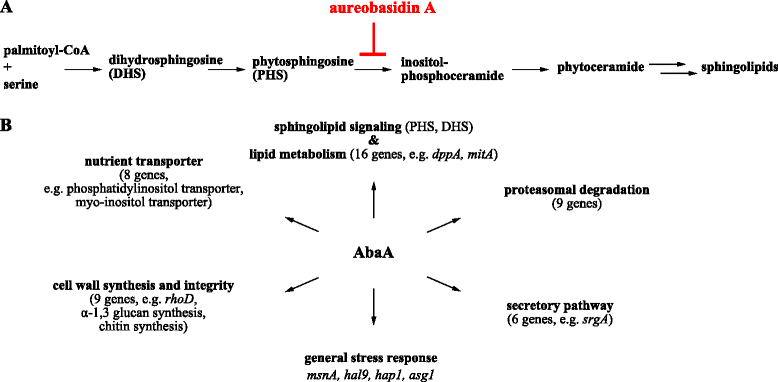

Supplement: Supplementary file 12 — Authors’ original file for figure 1 [file 40694_2014_5_MOESM12_ESM.gif]

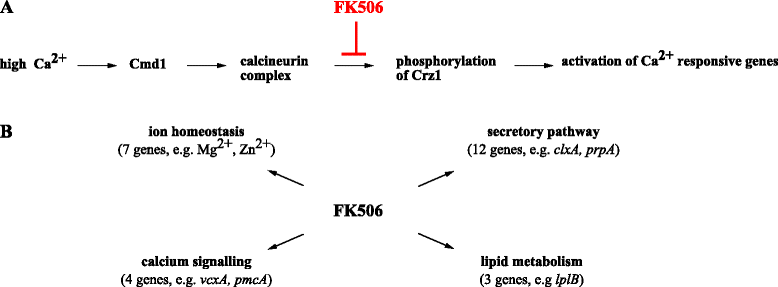

Supplement: Supplementary file 13 — Authors’ original file for figure 2 [file 40694_2014_5_MOESM13_ESM.gif]

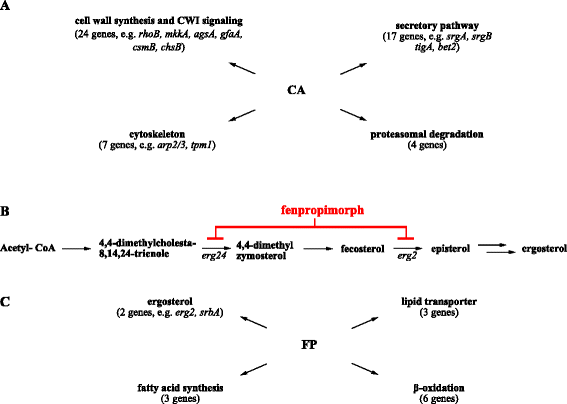

Supplement: Supplementary file 14 — Authors’ original file for figure 3 [file 40694_2014_5_MOESM14_ESM.gif]

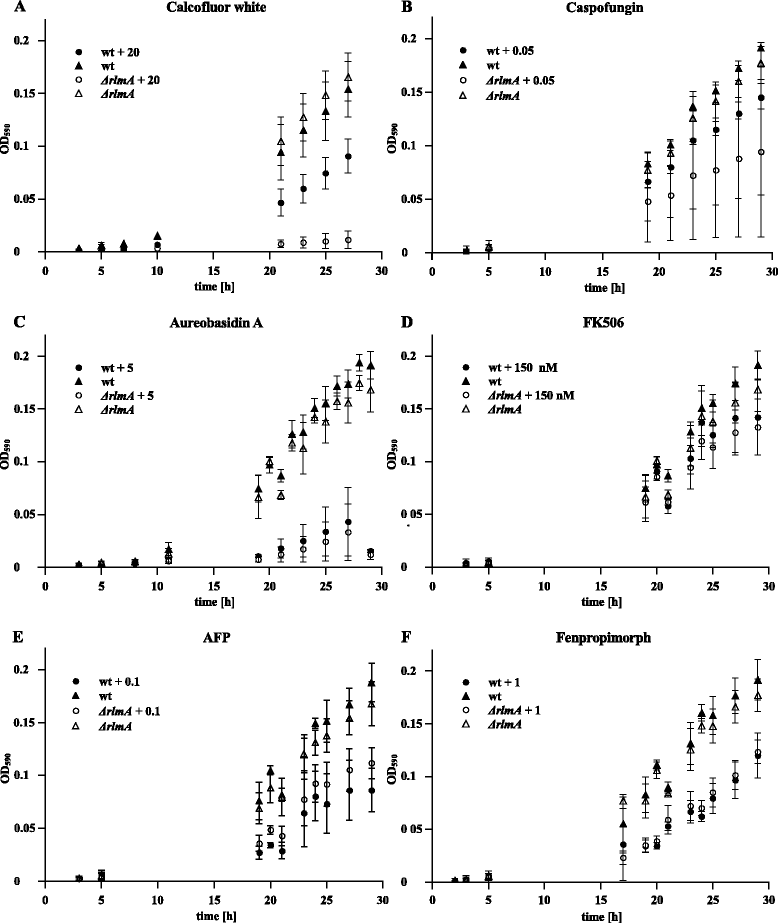

Supplement: Supplementary file 15 — Authors’ original file for figure 4 [file 40694_2014_5_MOESM15_ESM.gif]

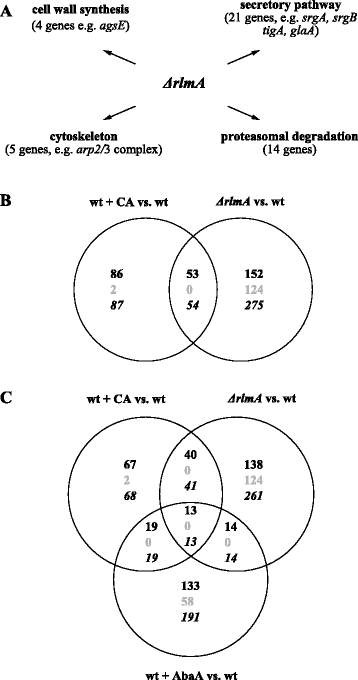

Supplement: Supplementary file 16 — Authors’ original file for figure 5 [file 40694_2014_5_MOESM16_ESM.gif]

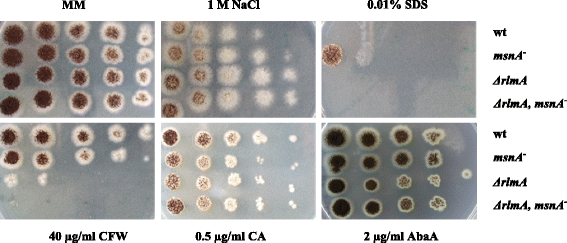

Supplement: Supplementary file 17 — Authors’ original file for figure 6 [file 40694_2014_5_MOESM17_ESM.gif]

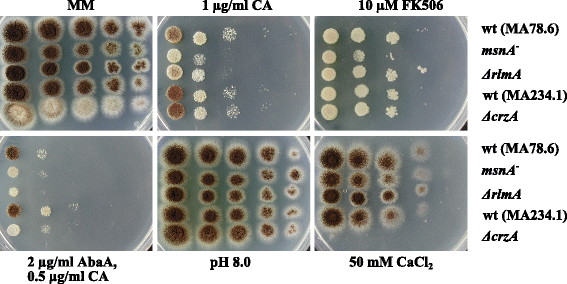

Supplement: Supplementary file 18 — Authors’ original file for figure 7 [file 40694_2014_5_MOESM18_ESM.gif]

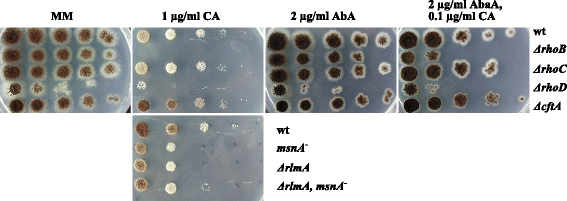

Supplement: Supplementary file 19 — Authors’ original file for figure 8 [file 40694_2014_5_MOESM19_ESM.gif]

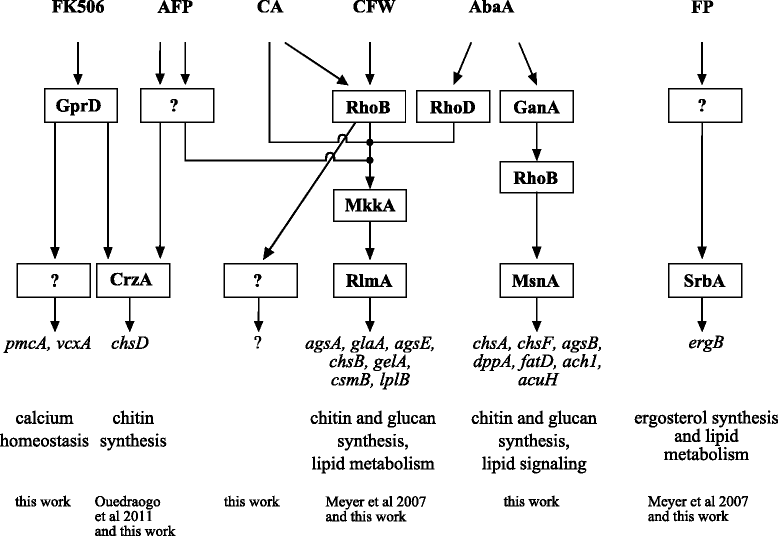

Supplement: Supplementary file 20 — Authors’ original file for figure 9 [file 40694_2014_5_MOESM20_ESM.gif]

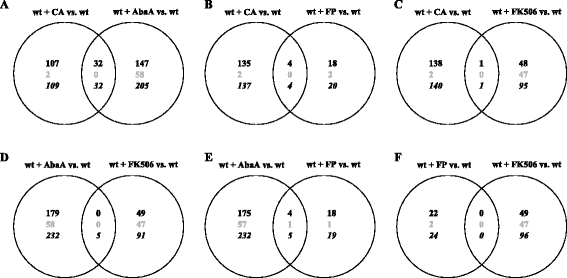

Supplement: Supplementary file 21 — Authors’ original file for figure 10 [file 40694_2014_5_MOESM21_ESM.gif]
